# Novel reconstruction procedure after total gastrectomy: clinical application of adjustable double-channel digestive tract reconstruction of jejunal interposition

**DOI:** 10.3389/fsurg.2025.1556183

**Published:** 2025-05-09

**Authors:** Guomin Zhou, Yu Yang, Jin Chen, Xiaoliang Tao, Hangyu Zhou, Rui Ming, Huaiwu Jiang

**Affiliations:** ^1^ Department of Gastrointestinal Surgery, The Affiliated Hospital of Southwest Medical University, Luzhou, Sichuan, China; ^2^ Department of Gastrointestinal Surgery, Mianyang 404 Hospital, Mianyang, Sichuan, China

**Keywords:** stomach neoplasms, total gastrectomy, reconstruction, Roux-en-Y anastomosis, nutritional status

## Abstract

**Background:**

Although diverse reconstruction techniques exist after total gastrectomy for gastric cancer, they have limited effectiveness. Adjustable double-channel digestive tract reconstruction of jejunal interposition (a-DJI) is an improved approach. This study compares this procedure with Roux-en-Y (RY) anastomosis to assess its clinical efficacy post-gastrectomy.

**Methods:**

The patients in this randomized controlled trial assigned patients to either the a-DJI group (experimental) or the RY group (control). Patients were followed for a total period of 1 year. Primary endpoints included perioperative indices; time to first flatus or feeding; complications (reflux esophagitis, dumping syndrome, and Roux retention syndrome); nutritional status (hemoglobin, total protein, albumin, vitamin D, and calcium); and dietary status.

**Results:**

From January 2021 to February 2023, 77 patients were enrolled with 39 and 38 patients in the a-DJI and RY groups, respectively. Reconstruction time, intraoperative blood loss, or time to first flatus/feeding did not differ significantly between groups (all *P* > 0.05). The a-DJI group had significantly lower rates of reflux esophagitis, dumping syndrome, and Roux retention syndrome (all *P* < 0.05) than those in the RY group. The RY group was more likely to consume <300 ml per meal and >5 meals per day than the a-DJI group (all *P* < 0.05). Body weight, hemoglobin, total protein, and albumin levels decreased lesser in the a-DJI group than those in the RY group (all *P* < 0.05). Vitamin D and calcium levels were higher in the a-DJI group than those in the RY group (all *P* < 0.05).

**Conclusion:**

The a-DJI is superior to RY in reducing complications and improving nutritional status in patients with gastric cancer after total gastrectomy.

## 1 Background

Gastric cancer is anticipated to be the fifth most common cancer and cause the fifth highest number of cancer-related deaths globally in 2022, according to the Global Cancer Statistics 2022 report from the International Agency for Research on Cancer of the World Health Organization (WHO) (1). The highest incidence of gastric cancer is in Eastern Asia, followed by Eastern and Central Europe (2). However, significant advancements have been made in the surgical treatment of gastric cancer over the past decades. The management of advanced gastric cancer involves radical surgical resection, with total or partial gastrectomy remaining the primary alternative for curative treatment in cases where endoscopic resection is infeasible (3, 4). The radical surgery outcomes have shifted from rapid improvement to a plateau of slow progress, despite the widespread use of standardized D2 radical gastrectomy (5). Gastrectomy damages the normal anatomy of the esophagus and stomach, impairing the functions of digestion and absorption, while undermining the original physiological pathways of the digestive tract. Consequently, nutritional status and quality of life of patients are significantly impacted by common complications such as reflux esophagitis, dumping syndrome, malabsorption, inadequate food intake, delayed gastric emptying, dyspepsia, weight loss, and anemia (6, 7). The postoperative quality of life in patients with gastric cancer is closely linked to the modes of digestive tract reconstruction (8). To mitigate postoperative complications, domestic and international researchers have developed and refined various reconstruction techniques, enhancing the postoperative quality of life that is a key focus of this study. Hongbo et al. reported that there are more than 70 surgical procedures for gastric cancer management following total or subtotal gastrectomy (9). No ideal, standardized, or optimal approach has been established globally, although each reconstruction method has its advantages and disadvantages. Therefore, a critical area of focus in gastric cancer surgery is innovative research on gastrointestinal (GI) reconstruction after gastrectomy.

Adjustable double-channel digestive tract reconstruction of jejunal interposition (a-DJI): The functional jejunal interposition (FJI) technique originally designed by Academician Hao Xishan was improvised by Huaiwu et al. (10). The a-DJI method includes features of jejunoileal interposition, Roux-en-Y (RY) anastomosis, and tab-type Braun anastomosis. There was excellent efficacy following preclinical application. To further validate this procedure, this study provides a detailed overview of the theoretical foundation and implementation of a-DJI, examines its clinical effects, and compares it with RY anastomosis.

## 2 Methods

### 2.1 Patients and randomization

This study selected patients diagnosed with gastric cancer requiring total gastrectomy between January 2021 and January 2023 at the Department of General Surgery, Mianyang 404 Hospital, Sichuan Province, based on the following inclusion criteria: (A) Presence of cancer of the esophagogastric junction or the upper or middle part of the stomach confirmed through preoperative gastroscopic pathology; (B) presence of stage II-III gastric cancer according to the WHO criteria (seventh edition of the American Joint Committee on Cancer TNM staging), with the potential for radical resection; (C) no history of other malignancies; (D) patients who signed informed consent; and (E) those with normal cardiac, pulmonary, renal, and hepatic functions. The exclusion criteria were as follows: (A) prior GI surgery; (B) underlying metabolic disorders; (C) pregnancy or lactation; (D) distant metastases (liver, lungs, bone, and other organs); and (E) other conditions deemed unsuitable for participation by the investigators.

The envelope method was used to randomly assign patients to the experimental or control group. The Biomedical Ethics Committee of Mianyang 404 Hospital approved this study involving human participants that was conducted in accordance with local regulations and institutional requirements (approval No. 2022-028). The contact information for the patient and two next of kin were recorded at discharge to facilitate further follow-up. A written informed consent was provided by all participants.

### 2.2 Surgical procedures

**Figure 1:**
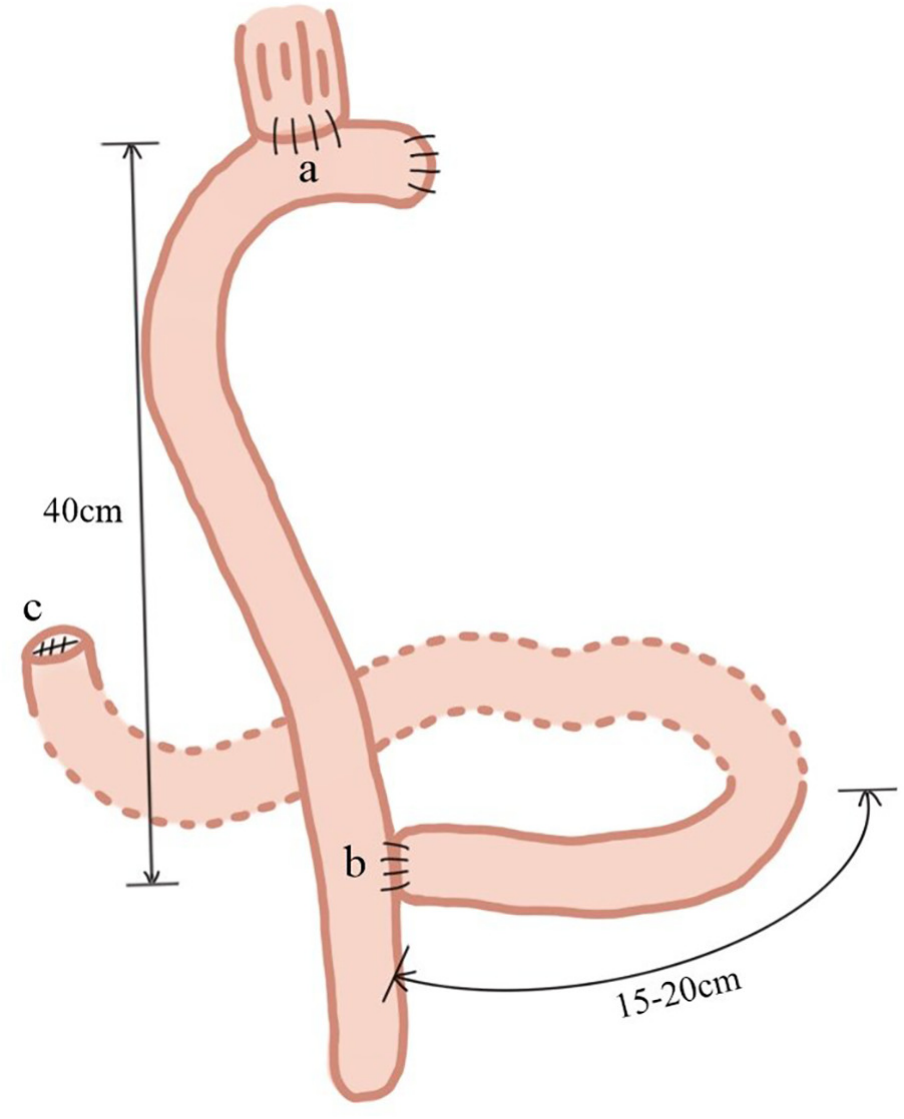
Roux-en-Y (RY) anastomosis. **(a)** The jejunum was transected approximately 15–20 cm from the ligament of Treitz, and the distal jejunum was anastomosed end-to-side with the lower esophagus, after lifting through the precolonial area; **(b)** the proximal jejunum was anastomosed with the distal jejunum end-to-side 40 cm below the esophagojejunostomy; **(c)** the proximal duodenum was closed.

GI reconstruction was performed using RY anastomosis in the control group. After a total gastrectomy, the jejunum was transected approximately 15–20 cm from the ligament of Treitz. The distal jejunum was anastomosed end-to-side with the lower esophagus after lifting through the precolonial area. The proximal jejunum was anastomosed with the distal jejunum end-to-side 40 cm below the esophagojejunostomy, followed by the closure of the proximal duodenum (Figure 1). In the experimental group, digestive tract reconstruction following total gastrectomy was performed using a-DJI as follows.

#### 2.2.1 The source and theoretical basis of a-DJI

The FJI method, proposed by Hao Xishan, allows food to pass through the duodenal route and preserves the continuity of the intestinal tract. It is a reasonable reconstruction alternative as it is simple to perform and easy to promote (11, 12). However, several shortcomings were identified during its application, which were as follows: (1) The duodenojejunal anastomosis, positioned at a right angle, is an end-to-side anastomosis, leading to delayed food passage. Although the output tab ligation site is only 2 cm from the anastomosis, food retention, spoilage, and bacterial proliferation occur because of the inevitable formation of a blind loop. This may be a significant pathological factor contributing to the 8.7% incidence of RY retention syndrome in this procedure. (2) The loss of pyloric and cardia function may still allow duodenal fluid reflux, although only a 4.3% incidence of reflux esophagitis has been reported, as pacing potentials controlled at the duodenal dominant site can be transmitted through the anastomosis, potentially exacerbating RY syndrome and contributing to reflux (13). (3) Blind segment is present in the afferent limb because this procedure ligates the jejunum 5-7 cm from the esophagus after the end-to-side anastomosis of the esophagus and jejunum in this procedure.

To further reduce complications, improvements were made to the FJI method based on its underlying construction theory. The modifications were as follows: (1) replacement of the ligation of efferent limb with partial reduction suture of the intestinal lumen. This modification aims to divert water from the dietary structure and low-quality coeliac of the patient into the adjustable channel, reducing postprandial satiety and increasing the volume of meals. In addition, when abnormal intestinal motility causes reflux of duodenal fluid, the flow is directed primarily through the regulated channel to the efferent limb, assisting in minimizing the occurrence of reflux esophagitis; (2) appropriately ensuring that the esophagojejunostomy and Braun anastomosis are free of tension by shortening the afferent limb. These enhancements retain all the advantages of the original method without increasing surgical complexity or time while addressing the following shortcomings: (i) elimination of the blind limb of the efferent limb. Food continues to pass mainly through the duodenum; however, a small amount, primarily fluids, passes through the partially narrowed regulatory channel into the distal jejunum, thus eliminating blind loops. Previous studies demonstrate that barium contrast predominantly enters the duodenum, with only a small amount passing through the narrowing, 1 and 6 months post-surgery. Two cases of efferent limb demonstrated complete opening of the narrowing after one month (14). (ii) The occurrence of reflux esophagitis can be further reduced as duodenal reflux fluid possibly passes through the partially narrowed regulatory channel. (iii) The input blind limbs are shortened as much as possible, considering the lack of jejunal angulation at the Treitz ligament and the absence of tension in the esophagojejunostomy and Braun anastomosis. This minimizes the phenomenon of blind limb observed in the original method.

#### 2.2.2 Specific implementation steps for a-DJI

**Figure 2:**
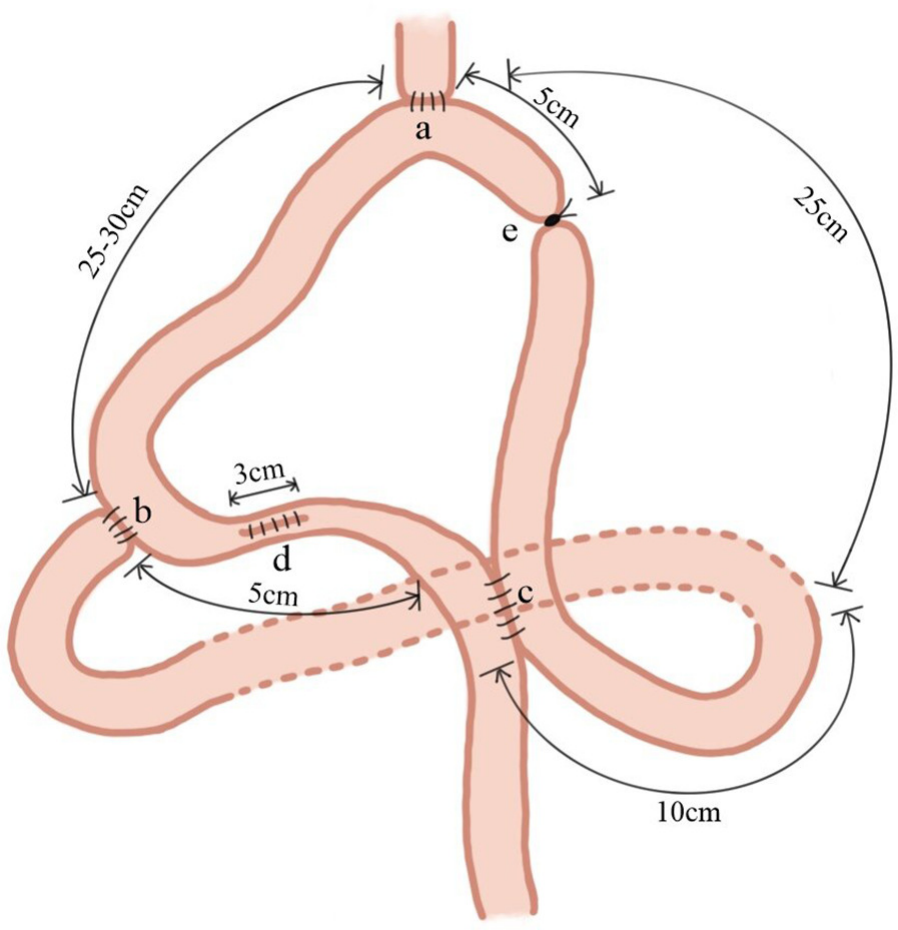
Adjustable double-channel digestive tract reconstruction of jejunal interposition **(a**-DJI). The jejunum, approximately 25 cm from the ligament of Treitz, was lifted through the precolonial area and anastomosed end-to-side with the lower esophagus; **(b)** the jejunum was anastomosed 25–30 cm distal to the initial anastomosis end-to-side to the duodenum; **(c)** the proximal jejunum was anastomosed side-to-side with the jejunum using a Braun anastomosis10 cm from the ligament of Treitz and 5 cm distal to the duodenal anastomosis; **(d)** the narrowing suture was placed between the duodenal and Braun anastomoses in the 5 cm segment of the jejunum. In addition, this section of the bowel was longitudinally incised for 3 cm; **(e)** the afferent limb between the esophageal and Braun anastomoses, measuring 5 cm, were moderately ligated or sutured using a thick wire.

The jejunum, approximately 25 cm from the ligament of Treitz, was lifted through the precolonial area after a total gastrectomy and anastomosed end-to-side with the lower esophagus. The jejunum was then anastomosed 25–30 cm distal to the initial anastomosis end-to-side to the duodenum. Subsequently, 10 cm from the ligament of Treitz, the proximal jejunum was anastomosed side-to-side with the jejunum using a Braun anastomosis, 5 cm distal to the duodenal anastomosis. The anastomosis was performed using a no. 26 anastomotic device. The anastomosis body was inserted into the lumen of the bowel to be narrowed. The narrowing suture was placed between the duodenal and Braun anastomoses in the 5 cm segment of the jejunum. In addition, this section of the bowel was longitudinally incised for 3 cm. After completing the anastomosis aforementioned, the bowel incision was sutured longitudinally to naturally complete the bowel constriction. Finally, to prevent recanalization of the collaterals postoperatively, the afferent limb between the esophageal and Braun anastomoses, measuring 5 cm, were moderately ligated or sutured using a thick wire (Figure 2).

### 2.3 Study design and endpoints

This study was a prospective, unblinded, and randomized controlled trial. All patients were routinely followed up at 1-year post-surgery. The postoperative complications, dietary status, and nutritional status were the primary endpoints. Dietary status was analyzed by the number of patients consuming <300 ml per meal and >5 meals per day. Based on the changes in plasma nutritional parameters, including hemoglobin (HGB), total protein (TB), albumin (ALB), vitamin D (VitD), and blood calcium (Ca), nutritional status was evaluated. Postoperative complications were reflux esophagitis, dumping syndrome, and Roux retention syndrome. Moreover, the secondary endpoints included operative time, intraoperative blood loss, the time to first flatus, and the time to the first feeding.

### 2.4 Statistical methods

Data were analyzed using SPSS 22.0 statistical software. Continuous data are presented as mean ± standard deviation (*x* ± *s*). The group comparisons were performed using an analysis of variance. Categorical data were compared using the *χ*² test, and the rank sum test was used for ordinal data. The statistical significance was set at *P* < 0.05.

## 3 Results

A total of 77 patients from January 2021 to January 2023, who met the inclusion and exclusion criteria were enrolled at the Department of General Surgery, Mianyang 404 Hospital, Sichuan Province. Of these, 39 and 38 patients were assigned to the a-DJI and RY groups, respectively. All patients were included in the study. No significant differences were noted between the two groups in baseline characteristics, including sex, age, body mass index, clinical stage, and preoperative nutritional status (Table 1).

**Table 1:** Comparison of baseline characteristics of patients between the two groups.

Characteristic	a-DJI (*n* = 39)	RY (*n* = 38)	*P*
Gender			
Male	27 (69.2%)	23 (60.5%)	0.424
Female	12 (30.8%)	15 (39.5%)	
Age (years)	56.4 ± 6.2	54 ± 6.0	0.253
BMI (kg/m^2^)	21.4 ± 2.7	21.1 ± 3.1	0.631
TNM stage			
Ⅱ	18 (46.2%)	16 (42.2%)	0.721
Ⅲ	21 (53.8%)	22 (57.9%)	
Weight (kg)	56.33 ± 6.32	55.79 ± 6.76	0.716
HGB (g/L)	108.18 ± 9.32	108.10 ± 8.60	0.695
TP (g/L)	49.92 ± 9.91	50.26 ± 3.86	0.702
ALB (g/L)	30.46 ± 2.13	30.24 ± 2.54	0.675
VitD (ug/ml)	37.04 ± 1.58	37.59 ± 1.14	0.161
Ca (mg/L)	60.41 ± 2.4	59.28 ± 1.65	0.056

### 3.1 Surgical and perioperative status

No perioperative deaths occurred in either group. No significant differences were observed regarding digestive tract reconstruction time, intraoperative blood loss, time to the first flatus, and time to the first feeding between the two groups (all *P* > 0.05; Table 2).

**Table 2:** Comparison of intraoperative conditions and perioperative recovery between the two groups.

Variable	a-DJI (*n* = 39)	RY (*n* = 38)	*P*
Reconstruction time (min)	47.2 ± 5.7	46.9 ± 5.0	>0.05
Intraoperative blood loss (ml)	261.2 ± 54.5	265.9 ± 56.4	
Time to first flatus (d)	3.0 ± 0.7	3.2 ± 0.6	
Time to first feeding (d)	4.8 ± 0.7	5.1 ± 0.9	

### 3.2 Postoperative complications

The a-DJI group had significantly lower incidences of reflux esophagitis, dumping syndrome, and Roux retention syndrome than the RY group (all *P* < 0.05; Table 3).

**Table 3:** Comparison of postoperative complications between the two groups.

Complication	a-DJI (*n* = 39)	RY (*n* = 38)	*P*
Reflux oesophagitis	3 (7.7%)	8 (21.1%)	<0.05
Dumping syndrome	3 (7.7%)	9 (23.7%)	
Roux retention syndrome	2 (5.1%)	12 (31.6%)	

### 3.3 Diet status

The RY group was more likely to consume <300 ml per meal and >5 meals per day than the a-DJI group (all *P* < 0.05; Table 4).

**Table 4:** Comparison of diet status between the two groups.

Diet status	a-DJI (*n* = 39)	RY (*n* = 38)	*P*
<300 ml per meal	5 (12.8%)	12 (31.6%)	<0.05
>5 meals per day	4 (15.4%)	12 (26.3%)	

### 3.4 Nutritional status

Body weight, HGB, serum TB, and ALB levels decreased lesser in the a-DJI group at 1 year postoperatively than that in the RY group (all *P* < 0.05; Table 5). The a-DJI group demonstrated significantly higher whole Ca and VitD levels than the RY group at 1 year postoperatively (all *P* < 0.05; Table 5).

**Table 5:** Reduction in weight, hemoglobin, total protein, and albumin between the two groups at 1 year postoperatively. Comparison of whole blood calcium and vitamin D levels between the two groups at 1 year postoperatively.

Variable	a-DJI (*n* = 39)	RY (*n* = 38)	*P*
Wight loss (kg)	4.0 ± 1.9	6.3 ± 3.9	<0.05
Haemoglobin loss (g/L)	7.7 ± 4.0	8.4 ± 3.0	
Total protein loss (g/L)	3.4 ± 1.6	4.3 ± 1.4	
Albumin loss (g/L)	3.3 ± 1.1	4.2 ± 0.8	
Calcium levels (mg/L)	47.81 ± 1.38	46.49 ± 0.78	
Vitamin D levels (ug/ml)	12.6 ± 1.35	11.82 ± 0.89	

## 4 Discussion

Surgical treatment for gastric cancer involves R0 resection of the tumor, dissection of lymph nodes, and digestive tract reconstruction. Therefore, healthcare providers are increasingly focused on managing postoperative complications, such as malnutrition, reflux esophagitis, dumping syndrome, and Roux retention syndrome to achieve long-term survival for patients (15, 16). Although several reconstruction techniques exist, none is universally optimal. Postoperative GI reconstruction for gastric cancer should expedite patient adaptation to the “stomach-less” state, promote a quick return to daily activities, and minimize postoperative discomfort using a straightforward procedure (16). RY anastomosis is commonly used for GI reconstruction because of its simplicity, fewer anastomoses, and ability to prevent bile and pancreatic reflux. However, complications like dumping syndrome or Roux retention syndrome cannot be prevented (17). In addition, RY reconstruction disrupts the continuity of the small bowel, and leaves the duodenum unused, often resulting in early postoperative bowel dysfunction (18). Zonca et al. prospectively evaluated the quality of life and functional emptying in patients with J-pouch reconstruction compared to RY anastomosis and found that emptying of the J-pouch followed a linear decreasing pattern, unlike the exponential pattern observed in the RY group. J-pouch reconstruction showed slower emptying but was associated with a higher quality of life than RY reconstruction (19). Therefore, a technique that preserves the duodenal channel and maintains intestinal neuromuscular function continuity could effectively resolve these challenges, leading to satisfactory outcomes. The a-DJI procedure is considered a common modification, combining features of jejunal interposition gastrostomy, RY anastomosis, and modified Braun anastomosis. By preserving the transduodenal pathway and maintaining jejunal continuity, it aligns with the physiological structure of the digestive tract, while providing a storage pouch function. This study assessed whether a-DJI had advantages over RY anastomosis in improving postoperative nutrition and quality of life in patients with gastric cancer.

No perioperative deaths were found in either group. No significant differences were found between groups in GI reconstruction time, intraoperative blood loss, time to the first flatus passage, or time to the first feeding. The findings of this study suggest that the a-DJI procedure does not increase the difficulties in surgery or perioperative risk. The incidence of reflux esophagitis, dumping syndrome, and Roux retention syndrome was lower in the a-DJI group than that in the RY group at 1 year postoperatively. Furthermore, the incidence of consuming <300 ml per meal and requiring >5 meals per day was also lower in the a-DJI group than that in the RY group. These findings suggest that GI reconstruction using the a-DJI is associated with greater meal capacity and lower meal frequency than that using RY anastomosis, further contributing to improved postoperative dietary status. Such improvements constitute as a basis for enhanced nutritional outcomes and quality of life. The a-DJI group exhibited a smaller decrease in body weight, HGB, serum TB, and ALB compared with the RY group at 1 year postoperatively. Additionally, whole Ca and VitD levels at 1 year postoperatively were higher in the a-DJI group than those in the RY group. These findings propose that the a-DJI procedure is more effective in maintaining postoperative nutritional status than RY anastomosis. Based on the aforementioned results, we propose the following reasons for the observed outcomes:

(1) Postoperative digestion with a-DJI mainly occurs through the duodenal channel; thus, preserving the physiological function of the duodenum. Food stimulation of the duodenal mucosa enhances the release of cholecystokinin and secretion of pancreatic fluid, promoting optimal mixing of food and digestive juices (20, 21). Previous studies have reported deficiencies of micronutrients following gastric surgery and results from bypassing the duodenum and proximal jejunum (22, 23). These are the primary sites for the absorption of iron, Ca, VitD, and other nutrients (24). In addition, Blonk et al. concluded that VitD deficiency was the most common, affecting 52% of patients after gastric resection (25). Therefore, these findings elucidate the superior nutritional status of the a-DJI group over the RY group in this study. The duodenum also assists in the maintenance of an alkaline environment, suppresses bacterial overgrowth, and supports mucosal growth (26, 27).
(2) The a-DJI preserves the continuity of the nerves and vasculature of the small intestine, unlike the RY group, maintaining the intestinal neuromuscular continuity, eliminating the effects of ectopic pacemakers, accelerating gastric emptying, and reducing the incidence of Roux retention syndrome (28). In contrast, jejunojejunostomy disrupts the physiological migratory motor complex in the RY group, with increased proximal jejunal motility potentially causing retrograde peristalsis and further contributing to Roux retention syndrome (29).
(3) The a-DJI procedure constricts the output limb lumen, facilitating food to pass through an adjustable double-channel and allowing gradient-based emptying. Most food passes through the duodenal channel, thereby reducing postprandial satiety and increasing single-meal intake, while water and less nutritious components are diverted through the secondary channel. In addition, the narrowed channel reduces tension at the duodenal anastomosis. The flow is directed into the secondary output limb, when abnormal motility causes duodenal reflux, reducing the risk of reflux esophagitis and Roux retention syndrome (30).
(4) The a-DJI procedure establishes a 25–30 cm jejunal loop between the esophageal and duodenal anastomoses, serving as a reservoir, partially compensating for the lost gastric capacity. This lowers the irritation on the esophagus from the alkaline digestive secretions, increases per meal volume while reducing meal frequency, and gradually normalizes the eating behavior postoperatively. This functioning is similar to the pyloric sphincter and effectively reconstructs a “functional pylorus”. Thus, further research is warranted to determine the optimal size of the regulatory channel opening to improve the “functional pylorus” effect and minimize postprandial satiety.

## 5 Conclusion

In patients with gastric cancer, the a-DJI is superior to RY anastomosis for digestive tract reconstruction after total gastrectomy. The a-DJI method is recommended for reconstruction in patients with advanced gastric cancer following total gastrectomy because it effectively reduces complications related to gastric absence and improves the nutritional status. This is our first international study that represents an initial exploration of the a-DJI procedure and a significant step in extending our research abroad. However, the study has several limitations. This study had a short follow-up duration and a small sample size, which limited the generalizability of the findings. Therefore, to assess long-term outcomes, further prospective studies with larger cohorts are required. The NutriOnc Research Group in Italy emphasizes that comprehensive nutritional assessment and implementation of clinical nutrition programs are critical for improving survival rates and quality of life in cancer patients. Future studies should prioritize the integration of nutrition specialists into multidisciplinary teams to enhance the delivery of nutritional care in oncology settings (31, 32). In addition to substantiating the effectiveness of the a-DJI procedure, we will also continue to explore long-term changes in micronutrient levels associated with it.

## Data Availability

The raw data supporting the conclusions of this article will be made available by the authors, without undue reservation.
